# Case Report: Bayesian Statistical Inference of Experimental Parameters via Biomolecular Simulations: Atomic Force Microscopy

**DOI:** 10.3389/fmolb.2021.636940

**Published:** 2021-03-10

**Authors:** Sotaro Fuchigami, Toru Niina, Shoji Takada

**Affiliations:** Department of Biophysics, Graduate School of Science, Kyoto University, Kyoto, Japan

**Keywords:** atomic force microscopy, probe tip, flexible-fitting, coarse-grained molecular simulation, CafeMol

## Abstract

The atomic force microscopy (AFM) is a powerful tool for imaging structures of molecules bound on surfaces. To gain high-resolution structural information, one often superimposes structure models on the measured images. Motivated by high flexibility of biomolecules, we previously developed a flexible-fitting molecular dynamics (MD) method that allows protein structural changes upon superimposing. Since the AFM image largely depends on the AFM probe tip geometry, the fitting process requires accurate estimation of the parameters related to the tip geometry. Here, we performed a Bayesian statistical inference to estimate a tip radius of the AFM probe from a given AFM image via flexible-fitting molecular dynamics (MD) simulations. We first sampled conformations of the nucleosome that fit well the reference AFM image by the flexible-fitting with various tip radii. We then estimated an optimal tip parameter by maximizing the conditional probability density of the AFM image produced from the fitted structure.

## Introduction

The atomic force microscopy (AFM) is a powerful tool for imaging the structures of molecules bound on surface at atomic resolution (Ando et al., 2001; Kodera et al., 2006; Kodera et al., 2010; Uchihashi et al., 2011; Casuso et al., 2012; Ando et al., 2013; Ando et al., 2014; Dufrêne et al., 2017). However, because the AFM measurement gives only height information and because biomolecular AFM measurements often provide medium-resolution images, one often seeks molecular structures that fit to the AFM image. The fitting of rigid molecules can be achieved simply by translating and rotating a given structure model to find the best match to the AFM image. On the other hand, when the target molecules are flexible, as are often the case for biomolecules, one needs to allow a structural change of the model upon superimposing. This so-called flexible-fitting has been successfully applied in the modeling based on the cryo-electron microscopy data by various methods, for example, the molecular dynamics flexible fitting (MDFF) method and its extension (Trabuco et al., 2008; McGreevy et al., 2016; Singharoy et al., 2016), the correlation-coefficient-based method (Orzechowski and Tama, 2008), and CryoFold (Shekhar et al., 2020). The flexible-fitting can be realized by using molecular dynamics (MD) simulations, where a fitting score is integrated with the standard molecular mechanics force field. Recently, we developed a flexible-fitting MD method for finding molecular structures that fit the AFM image (Niina et al., 2020).

Generally, these fitting processes require knowledge on parameters that characterize the measurement, of which values are often unknown a priori. Therefore, one challenge is to infer these parameters, simultaneously finding the target molecular structures. In the case of the AFM measurement, such parameters include the size and the shape of the probe tip, which are usually unknown, but strongly affect the resulting images.

**FIGURE 1:**
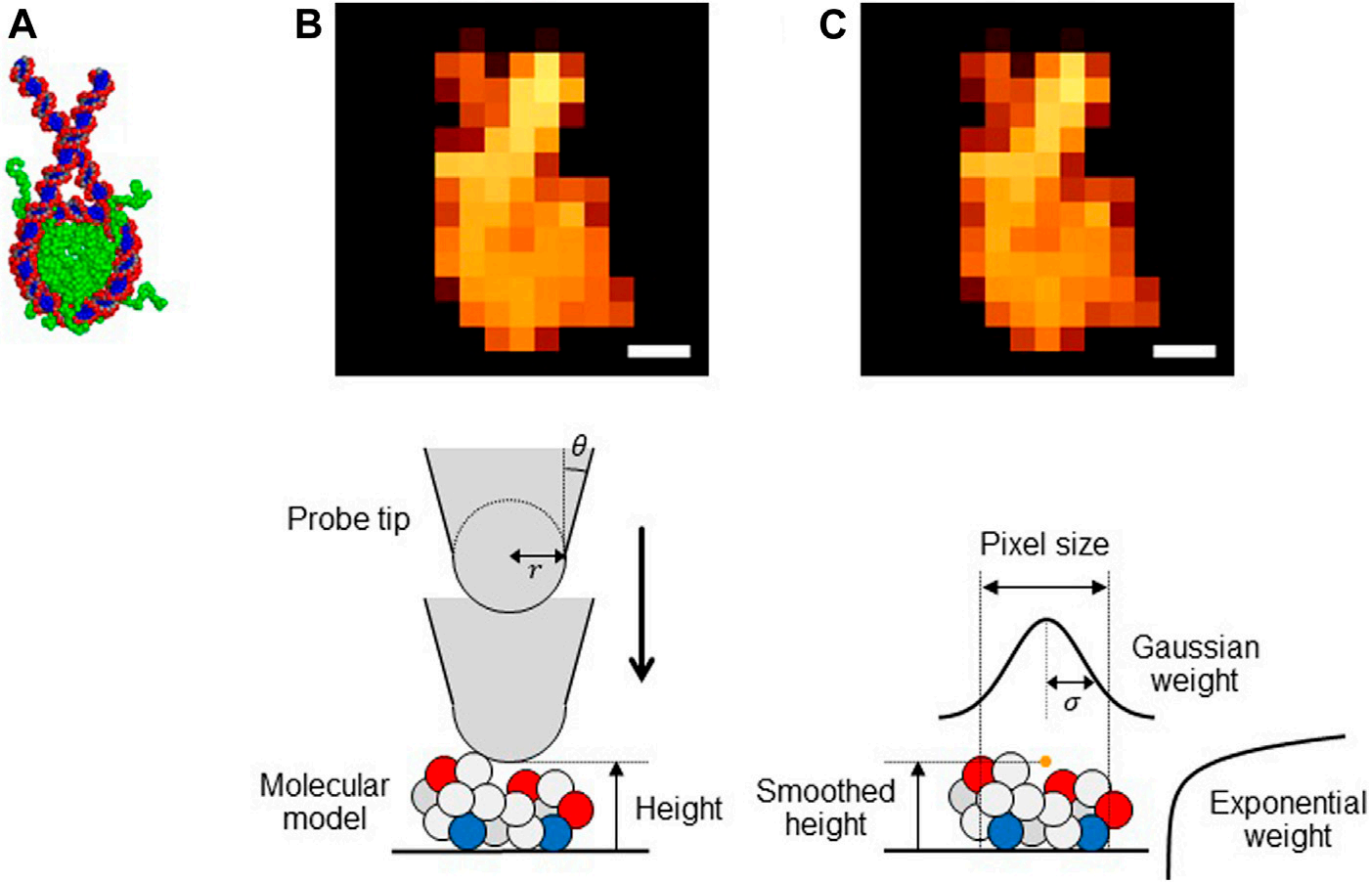
**(A)** Structure of a nucleosome used in this study. **(B** **and** **C)** Pseudo-AFM images of nucleosome (Scale bar: 5 nm) generated **(B)** by the collision detection method and **(C)** by the smoothed method **(upper)** and schematic views of these methods **(lower)**.

In this brief report, we focus on the inference of the radius of the AFM probe tip, assuming that it has the spherical shape. Using a Bayesian statistical inference approach, we examine feasibility of the inference of the AFM probe tip radius via flexible-fitting process. We chose a nucleosome as the test molecule (Figure 1A). The nucleosome consists of a histone octamer and a duplex DNA of 223 base pairs, and the initial structure was modeled using crystal structure (PDB ID: 3LZ0), as described in our previous work (Niina et al., 2017; Fuchigami et al., 2020).

## Methods

### Pseudo-AFM Image Generating Method

The collision detection method: The collision-detection method is to generate a pseudo-AFM image for a given molecular structure. It assumes a simple geometry of the probe tip; the cone-shape with its terminus being a sphere (Figure 1B, lower). Thus, the tip can be characterized by the two parameters, the tip radius R and the apex angle θ. In this study, we fix the latter as θ=10 degree. For each pixel, we calculate the height of the bottom of the sphere at which a steric collision between the tip end and atoms in the molecular models occurs (Figure 1B).

The collision-detection method is available as a tool “afmize” (http://doi.org/10.5281/zenode.3362044). The BioAFMviewer, which provides the pseudo-AFM image via a similar method, is also available (Amyot and Flechsig, 2020).

The smoothed method: While the collision detection method offers perhaps the most straightforward way to generate a pseudo-AFM image from a given structure model, the method does not give a form differentiable with respect to atomic coordinates of target molecules. This precludes its usage in the flexible-fitting MD since MD simulations require the force calculations that include the differentiation of the fitting score. To this end, we previously proposed a differentiable proxy of the collision detection method, which we call the smoothed method. For a target biomolecule represented by its coordinate $\left(x_{j},\;y_{j},\;z_{j}\right)$ and radius $r_{j}$ for the *j*-th particle (j=1,…, N), we define the smoothed height $H_{p}^{\left(sim\right)}$ at the *p*-th pixel $\left(x_{p},\;y_{p}\right)$ as

$$H_{p}^{\left(sim\right)}\;=\gamma \log\left\{1+\sum_{j=1}^{N}\exp\left[\frac{-{\left(x_{j}\;-\;x_{p}\right)}^{2}-\;{\left(y_{j}\;-\;y_{p}\right)}^{2}}{2\sigma^{2}}\right]\exp\left(\frac{z_{j}+r_{j}}{\gamma }\right)\right\}.$$ (1)

We assumed xy-plane as the AFM stage and thus that the *z*-coordinate represents the height of the target molecule. This function contains smoothing in *xy*-directions (characterized by σ), as well as the smoothing in *z*-direction (characterized by γ) (Figure 1C).

Importantly, in the smoothed method expression, the tip radius R does not appear directly. Instead, we previously showed that the optimal value of σ well correlates with the tip radius R. Thus, by changing σ, we can effectively generate pseudo-AFM images of different R’s.

We note that a smaller γ corresponds to a closer approximation to the collision-detection method, but a too small γ leads to a sharp change in the force and thus to the instability of MD simulations. In this study, we fix γ=0.1 nm.

The smoothed method is also available in “afmize” (http://doi.org/10.5281/zenode.3362044).

### Flexible-Fitting Molecular Dynamics Simulation

Flexible-fitting MD simulations utilize the total potential energy function, $V_{total}=V_{phys}+V_{AFM}$. Here, $V_{phys}$ represents the physical interaction of the target molecules as well as their interaction with the surface, the latter of which is modeled as a simple Lennard-Jones potential along the z axis. The second term is defined as $V_{AFM}\left(R\right)=\;\kappa Nk_{B}T\left[1\;-\;c.c.\left(R\right)\right]$, where $k_{B}$ is the Boltzmann constant, T is the temperature (300 K in this study), κ is a dimensionless parameter that controls the strength of the bias (unity in this study), and R is the coordinate of a simulated molecule. (Niina et al., 2020). The c.c.(R) is the modified correlation coefficients between the pseudo-AFM image $H_{p}^{\left(sim\right)}\left(R\right)$ of the simulating structure R and the real experimental AFM image $H_{p}^{\left(\exp\right)}$, defined as

$$c.c.\left(R\right)\;=\frac{\sum_{p\;\in \;pixels}H_{p}^{\left(\exp\right)}H_{p}^{\left(sim\right)}\left(R\right)}{\sqrt{\sum_{p\;\in \;pixels}{\left(H_{p}^{\left(\exp\right)}\right)}^{2}}\sqrt{\sum_{p\;\in \;pixels}{\left(H_{p}^{\left(sim\right)}\left(R\right)\right)}^{2}}}\;.$$ (2)

See the reference (Niina et al., 2020) for more details.

### Coarse-Grained Molecular Dynamics Simulation

In this study, as the physical interaction part of $V_{phys}$, we used a well-tested coarse-grained model for proteins and DNAs, which have been extensively used in many recent studies, including simulations for nucleosomes (Niina et al., 2017; Brandani et al., 2018; Tan and Takada, 2020). Briefly, proteins are represented by chains of beads, each of which represents an amino acid. For DNAs, each nucleotide is approximated by three particles, each representing sugar, phosphate, and base groups. We used the energy function AICG2+ for proteins (Li et al., 2014), 3SPN.2C for DNA (Freeman et al., 2014). Protein-DNA interactions are modeled by the excluded volume term and the electrostatic interaction (Fuchigami et al., 2020). See the reference (Fuchigami et al., 2020) for more details.

All the simulations were performed by CafeMol (Kenzaki et al., 2011). Specifically, the current simulation setup is provided as an example of the flexible-fitting MD simulation in the CafeMol package.

## Results

### Relationship Between the Tip Radius and the Parameter σ

**FIGURE 2:**
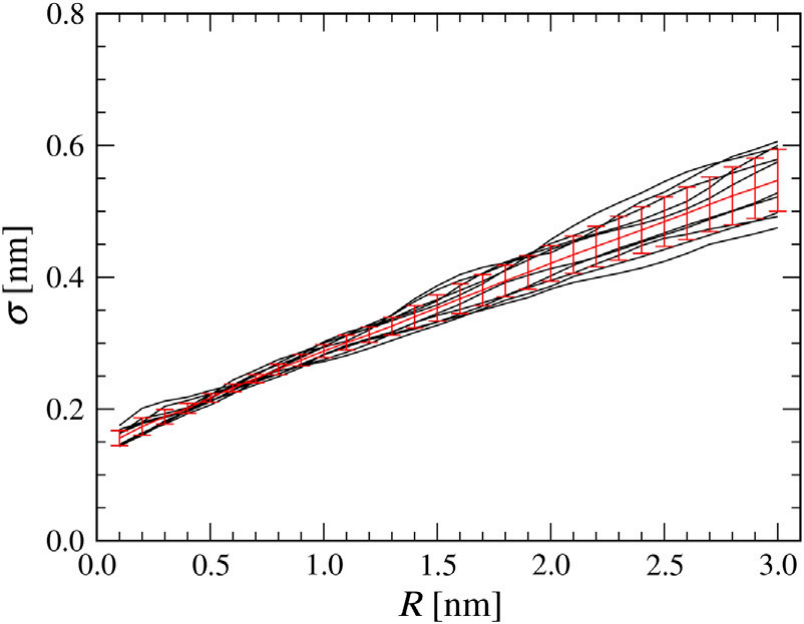
Relationship between a tip radius R in the collision detection method and a parameter σ in the smoothed method. The results from ten structures of nucleosome are shown in black lines, and the mean with the standard deviation for each tip radius R are shown in red.

Before performing the flexible-fitting MD simulations, we quantify the relation between the AFM probe tip radius in the collision detection method and the σ parameter used in the smoothed method. For ten distinct structures of nucleosomes obtained by coarse-grained MD simulations, we first generated pseudo-AFM images by the collision detection method with various tip radii R’s, ranging between 0.1 and 3.0 nm (with 0.1 nm increment). For each R value, we searched the optimal σ value with which the smoothed method gives the maximum correlation coefficient (c.c.) with the image generated by the collision detection method (Figure 2). We found that, on average, the optimal σ value for each nucleosome structure linearly increases with the tip radius R. In addition, the optimal σ value depends, albeit modestly, on the nucleosome structure. Especially, for larger tip radius, the optimal σ value varies structure by structure more significantly (Figure 2). For each tip radius R, we quantified the optimal σ value by its mean 〈σ(R)〉 and the standard deviation Δσ(R) of the ten samples (Supplementary Table S1).

Using the Bayes’ theorem, we can estimate the probability density to have the tip radius R for a given σ as,

$$P\left(R|\sigma \right)=\frac{P\left(\sigma |R\right)P\left(R\right)}{\sum_{R}P\left(\sigma |R\right)P\left(R\right)}$$ (3)

where we assumed the discrete representation of the tip radius (0.1 nm increment). Without any prior knowledge, we assume P(R) is constant over the range between 0.1 and 3.0 nm. We also assume that P(σ|R) is well approximated by the normal distribution with the mean 〈σ (R)〉 and the standard deviation Δσ(R), i.e., $P\left(R|\sigma \right)=N\left(\langle \sigma \;\left(R\right)\rangle ,\Delta \sigma \;{\left(R\right)}^{2}\right)/\sum_{R}N\left(\langle \sigma \;\left(R\right)\rangle ,\Delta \sigma \;{\left(R\right)}^{2}\right)\;$. The resulting conditional probability density is given in Supplementary Table S2.

### Bayesian Statistical Inference of the Tip Radius Using Flexible-Fitting MD Simulation

To examine the Bayesian statistical inference of the tip radius unambiguously, we need a test system for which we know the “correct” tip radius. To this end, in this study, we performed the so-called twin experiment. We first prepared a reference AFM image of the nucleosome using the collision detection method with the tip radius of 1.0 nm. This AFM image serves as the “experimental AFM image” in this study. Thus, the “correct” tip radius in this study is 1.0 nm.

**FIGURE 3:**
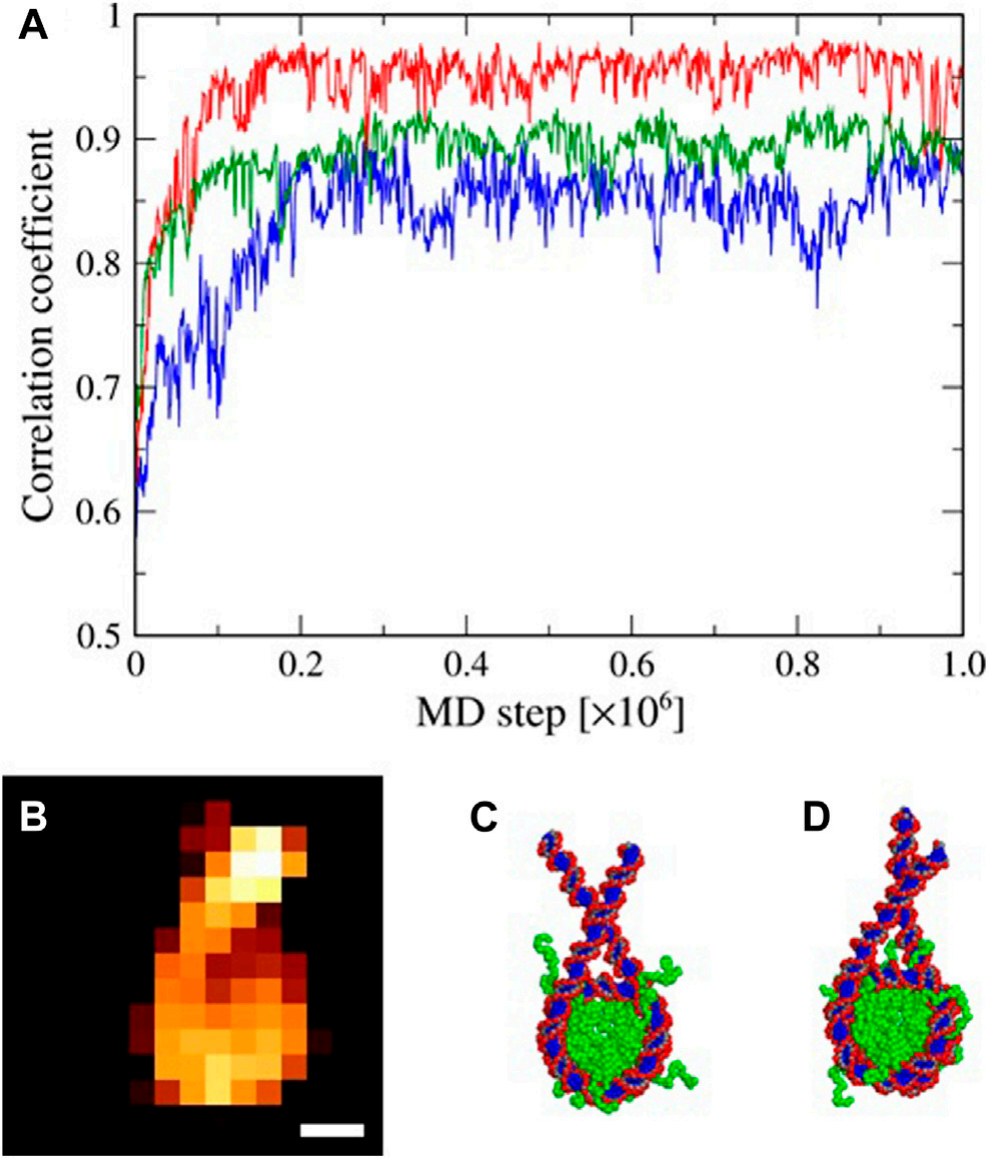
Flexible-fitting MD simulations. **(A)** Representative time series of the correlation coefficient (c.c.) of pseudo-AFM image of the simulated structure with the “experimental” AFM image. Shown here are results from three different σ values, 0.1, 0.3, and 0.5 nm in blue, red, and green, respectively. **(B)** The synthetic-AFM image used as an “experimental” AFM image in the flexible-fitting. Scale bar: 5 nm. **(C)** The nucleosome initial structure. **(D)** The simulated structure with the highest c.c..

For the experimental AFM image (Figure 3B), starting from a different structure (Figure 3C), we performed 10^6^-step flexible-fitting MD simulations of the nucleosome system with various values of σ ranging from 0.10 to 0.50 nm (with the 0.01 nm increment) (exemplified in Figure 3A). We repeated the same simulations ten times with different random numbers for each value of σ. The time series of the c.c. between the experimental and the simulated AFM images clearly show that the c.c. stochastically increases as a function of time, reaching to a plateau in all the simulations, successfully obtaining well-fitted structures (Figure 3D). The averaged plateau value, however, changes depending on the value of σ (Figure 3A).

**FIGURE 4:**
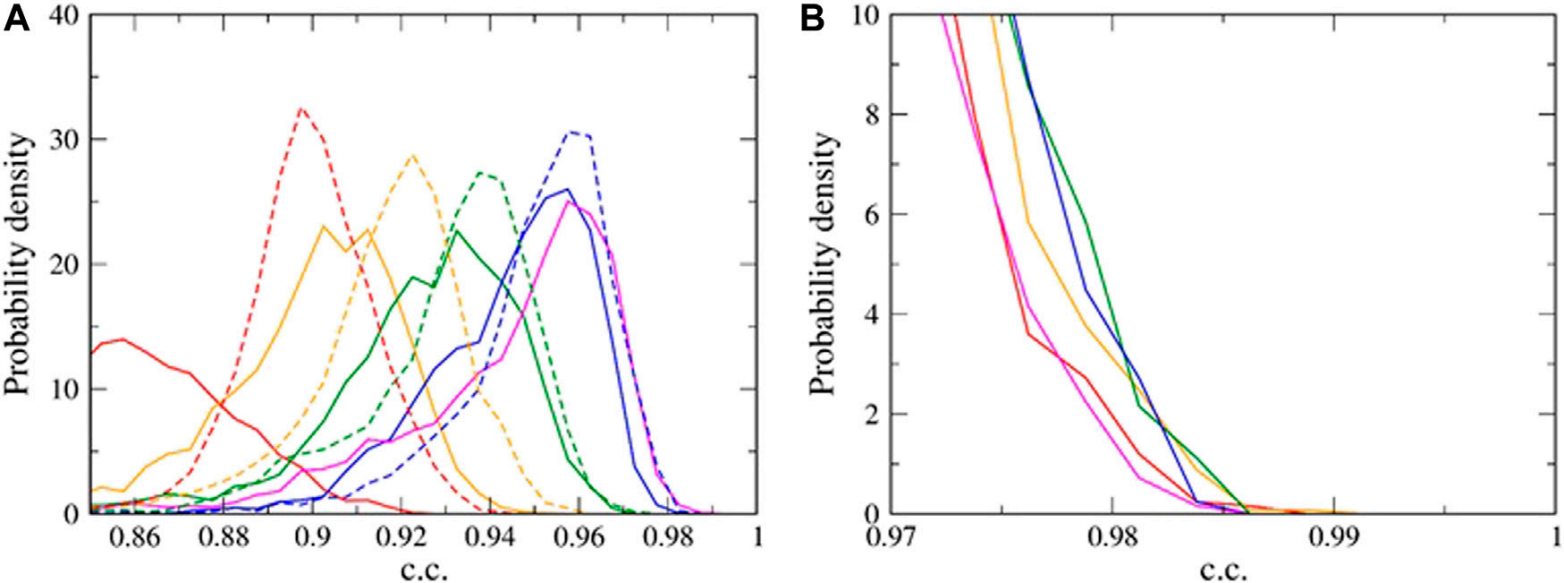
The probability density distributions of the correlation coefficient (c.c.) for representative σ values. **(A)** The overall distribution. The σ in nm unit are 0.1 (red solid), 0.15 (orange solid), 0.20 (green solid), 0.25 (blue solid), 0.30 (magenta solid), 0.35 (blue broken), 0.40 (green broken), 0.45 (orange broken), and 0.50 (red broken). **(B)** A close-up view at a high c.c. range. The σ in nm unit are 0.30 (red), 0.31 (orange), 0.32 (green), 0.33 (blue), and 0.34 (magenta).

To quantify the difference in the reached correlation coefficient (c.c.), we then estimated the probability density distributions of the c.c. for different σ values using the latter half of the time series data, i.e., totally 5,000 snapshots (representative cases shown in Figure 4). For example, a very small σ, such as 0.10 nm, resulted in the distribution with low c.c.’s, suggesting a clearly poor fitting. A rather large σ, such as 0.5 nm, also gave a poor fitting. In between the two limiting cases, there exists an optimal value of σ. Our previous studies indicated that the mean, or the median, of the c.c. distribution is not a good indicator (Niina et al., 2020). Instead, we need to focus on the upper tail of the distribution of c.c.. Among the nine cases in Figure 4A (between 0.1 and 0.5 nm, with the 0.05 nm increment), we found σ = 0.3 nm the optimal value.

Looking into a finer scale (0.01 nm increment) shown in Figure 4B, we found the highest c.c. value 0.989 at σ = 0.31 nm. From the values of conditional probability density shown in Supplementary Table S2, it is found that the most probable value of tip radius R is 1.2 nm, slightly larger than the “correct” tip radius 1.0 nm. This difference between the correct and the estimated tip radii is inevitable and acceptable because AFM image is insensitive to sub-nanometer-order difference in tip radius. Indeed, pseudo-AFM images generated using 1.0 nm-radius tip shows a high c.c. of 0.99 or higher with the images generated using tip with radii of 0.7, 0.8, 0.9, 1.1, 1.2, and 1.3 nm.

To test the statistical significance of the estimation of σ value, we performed a simple bootstrap test. We first prepared all patterns of combinations of five samples taken from ten highest c.c. values obtained from ten flexible-fitting MD simulations with the same σ value. The number of combination patterns is _10_C_5_ = 252. In each combination pattern, the highest c.c. value in the five values was used as the estimated value. In this way, 252 estimated highest c.c. values were obtained for each of seven σ values, σ=0.28, 0. 29, 0.30, 0.31, 0.32, 0.33, and 0.34  nm. Then, we performed all-to-all pairwise comparison of 252 estimated highest c.c. values for σ=0.31 nm with 252 estimates for the other six σ values. The σ=0.31 nm exhibited higher values against for σ=0.28, 0. 29, 0.30, 0.32, 0.33,and 0.34  nm with the probabilities 0.956, 0.998, 0.723, 0.677, 0.986, and 0.957, respectively. Thus, the σ value can be estimated as 0.31±0.01 nm. Note that the bootstrap test was performed with five c.c. values, whereas the real estimate used ten c.c. values, making the latter somewhat more reliable.

## Discussion

In this brief report, we investigated the statistical inference of the AFM probe tip radius via flexible-fitting MD simulations. First, we statistically characterized the relationship between the tip radius appeared in the collision detection method and the σ value used in the smoothed method. Then, with various σ values, we repeated the flexible-fitting MD simulations to the pre-defined AFM image of a nucleosome structure. Based on the upper tail part of the probability density distribution of the correlation coefficient, we found an optimal value of σ, as well as a well-fitted structure, for the given AFM image. Combining it with the above-mentioned relationship between the tip radius and the σ value, we could infer the probe tip radius, with reasonable accuracy.

As shown in Figure 2, a good linear correlation is observed between a tip radius R and a parameter σ. In this case, therefore, the simple linear regression-based method could also work well to infer a tip radius from a σ value.

One possible limitation in the current approach is in the treatment of flexible regions of proteins. In the current test system, the nucleosome contains the histone octamer, which contain rather long disordered tails. Usually, the AFM measurements do not clearly detect the configuration of the histone tails. On the other hand, our collision detection method and the smoothed method detect such flexible regions in the same way as the well-folded regions. While fitting of the well-folded regions is realized in the current simulations, disordered tail configurations were not well fitted, which could cause some discrepancy in the tip radius inference.

While the current approach can infer the tip radius with reasonable accuracy, there can be room for improvement especially in terms of the estimate efficiency. The current approach needs to repeat the flexible-fitting MD with various σ values. This is feasible for the inference of one parameter, and can be accelerated by using an efficient search method, for example, a two-stage searching method with coarse and fine increments. But it is nearly impossible for the case of many unknown parameters. In the context of AFM measurement, there can be other relevant parameters, such as the apex angle of the probe cone. For these cases, some improvement may be necessary. Since inference of the experimental parameters in the flexible-fitting MD is quite a computationally demanding process, we may better perform similar inference within the rigid-body fitting process. In addition, relationship between the tip radius and the σ value might be somewhat system-dependent. It would be confirmed by performing the same analysis using other molecules.

## Data Availability

The raw data supporting the conclusions of this article will be made available by the authors, without undue reservation.
